# The Microenvironment Is a Critical Regulator of Muscle Stem Cell Activation and Proliferation

**DOI:** 10.3389/fcell.2019.00254

**Published:** 2019-10-29

**Authors:** John H. Nguyen, Jin D. Chung, Gordon S. Lynch, James G. Ryall

**Affiliations:** Department of Physiology, Centre for Muscle Research, The University of Melbourne, Melbourne, VIC, Australia

**Keywords:** injury, repair, skeletal muscle, metabolism, metabolic reprogramming

## Abstract

Skeletal muscle has a remarkable capacity to regenerate following injury, a property conferred by a resident population of muscle stem cells (MuSCs). In response to injury, MuSCs must double their cellular content to divide, a process requiring significant new biomass in the form of nucleotides, phospholipids, and amino acids. This new biomass is derived from a series of intracellular metabolic cycles and alternative routing of carbon. In this review, we examine the link between metabolism and skeletal muscle regeneration with particular emphasis on the role of the cellular microenvironment in supporting the production of new biomass and MuSC proliferation.

## Introduction

Skeletal muscle has a remarkable potential to regenerate following injury, a property conferred by a population of local somatic stem cells termed muscle stem cells (MuSCs). In response to damage or trauma, local MuSCs are quickly activated and undergo extensive rounds of proliferation, differentiation, fusion and maturation in order to repair and/or replace damaged tissue (Charge and Rudnicki, 2004; Relaix and Zammit, 2012; Wosczyna and Rando, 2018). The extent of the MuSC response varies depending on the severity of the initial insult; ranging from a minor strain to major trauma from laceration, ischemia-reperfusion, or myotoxicity. Importantly, the proliferative response of MuSCs to injury is dependent on the capacity of these cells to double their cellular content, requiring synthesis of new biomass in the form of nucleotides, phospholipids, and non-essential amino acids (NEAA) (Koopman et al., 2014; Hosios et al., 2016). Synthesis of these molecules requires a ready supply of carbon-based precursors, satisfied by nutrients in the local extracellular tissue environment.

In this review, we will discuss recent findings linking cellular metabolism and the extracellular environment to cell division, and how efficient carbon routing is critical for MuSC proliferation and successful skeletal muscle regeneration. First, we will provide a brief overview of skeletal muscle regeneration.

## An Overview of Skeletal Muscle Injury and Repair

Muscle injuries can result from physical insults, diseases, toxins, and following ischemia (Souza and Gottfried, 2013). Although mechanical damage to muscle fibers can occur with daily activities and exercise, more severe injury can result in an irreversible loss of functional capacity. These more severe injuries include; contusion, strain, or laceration (Järvinen et al., 2005). Contusions are the most common mechanical insult, arising from a blunt, non-penetrating force that can rupture blood vessels and cause hematomas (Crisco et al., 1994). Strain injuries arise from high external loads that overstretch activated myofibers, damaging their structure and in more severe cases, the interconnections between muscle-tendon and tendon-bone (Nikolaou et al., 1987). A higher risk of strain injuries comes with advancing age or diseases that render muscles more vulnerable to damage (Lynch, 2004; Baker, 2017). Laceration is caused by a penetrative or crushing force, often leading to tissue loss and formation of scar tissue (Garrett et al., 1984). These severe muscle injuries require longer periods of regeneration and carry an increased risk of incomplete muscle repair.

Muscle regeneration is complex, requiring the coordinated activity of inflammatory cells, fibroblasts, mesenchymal cells, and MuSCs to ensure complete restoration of vasculature, nerves, and myofibers (Christov et al., 2007; Dumont et al., 2015). As mature myofibers are post-mitotic, muscle regeneration is dependent on an adequate population of viable MuSCs.

**FIGURE 1:**
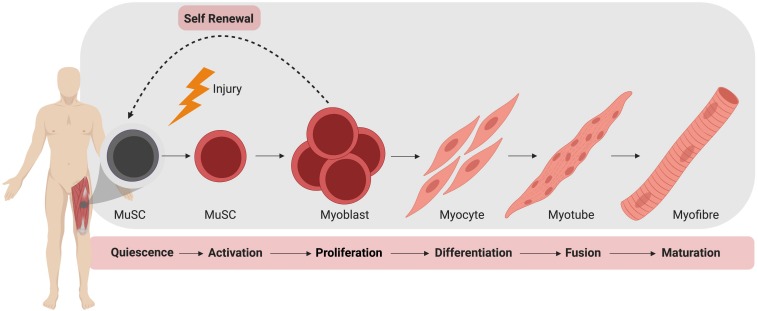
Muscle stem cell (MuSC) mediated skeletal muscle regeneration. Following injury, quiescent MuSCs are activated and undergo rapid proliferation, followed by differentiation into myocytes, which fuse and mature to generate new muscle fibers.

In the absence of injury, MuSCs typically exist in a quiescent state outside of the cell cycle, residing between the plasma membrane of a myofiber and the basement membrane (Mauro, 1961). During homeostasis, MuSCs do not actively proliferate and typically account for 2–10% of myonuclei, depending on age, sex, and muscle type (Dumont et al., 2015). Upon activation, MuSCs produce a progeny of myogenic cells that can differentiate, culminating in the formation of mature muscle fiber (Figure 1). During this process, MuSCs typically become specified to the myogenic lineage after activation and then undergo multiple rounds of proliferation to generate sufficient myonuclei to support protein synthesis and mature muscle formation (Bischoff, 1990). These proliferating myogenic precursors (myoblasts) then exit the cell cycle and terminally differentiate to myocytes which subsequently fuse to form myotubes. Muscle regeneration is completed through further rounds of myoblast fusion and muscle fiber maturation (Knudsen and Horwitz, 1977). Importantly, a small subpopulation of myoblasts return to quiescence so as to restore the MuSC pool.

It is important to note the balance between differentiation and maintenance of the MuSC pool during regeneration. While the majority of MuSCs will undergo activation and proliferation after injury, a sub-population of MuSCs must be maintained for regeneration of subsequent injuries (Collins et al., 2005; Sacco et al., 2008). To maintain this population, MuSCs may undergo symmetric or asymmetric division, respectively, producing either two identical daughter cells or both a single undifferentiated daughter cell and a committed myogenic precursor (Dumont et al., 2015). Control of differentiation versus self-renewal of MuSCs is governed by transcription factors, epigenetics and signaling pathways, and achieving an appropriate balance is key to sustaining muscle plasticity and regenerative capacity (Yin et al., 2013).

When muscle fibers are damaged, MuSCs are activated by both physical and chemical signals. With severe mechanical muscle injuries, ruptured blood vessels cause local hematoma and affected myofibers seal off damaged portions of the cell to prevent the spread of necrosis (Carpenter and Karpati, 1989; Hurme et al., 1991). Resident mast cells release cytokines that increase blood flow and attract circulating inflammatory cells. These cells phagocytose necrotic debris and release cytokines that promote survival of damaged cells (Pillon et al., 2012). The rate of muscle regeneration is also highly dependent on angiogenesis, as blood vessels and endothelial cells supply nutrients and mitogens for MuSC growth (Järvinen, 1976; Christov et al., 2007). Direct damage to the basal lamina or expression of matrix metalloproteinase stimulated by nitric oxide release, may further release trapped growth factors in the extracellular matrix that also encourage activation of MuSCs (Dimario et al., 1989; Tatsumi, 2010). These signals cause MuSCs to leave quiescence, migrate to the site of injury and begin proliferating.

After sufficient proliferation, myocytes fuse to form new, immature myotubes or fuse to existing injured fibers (Knudsen and Horwitz, 1977). Immature myotubes are centrally nucleated and gain functional capacity as they increase in size and express contractile proteins. This fusion is supported by the infiltrated inflammatory cells, as they adopt an anti-inflammatory phenotype that prevents excess damage to remaining healthy tissue (Arnold et al., 2007). The maturation and hypertrophy of myotubes is also supported by the release of insulin-like growth factor 1 (IGF1) from anti-inflammatory M2 macrophages, stimulating protein synthesis through the activation of the Akt-1/mTOR signaling pathway (Rommel et al., 2001; Park and Chen, 2005).

The role of metabolism and the local microenvironment in muscle regeneration has recently received attention (Ryall et al., 2015a; Pala et al., 2018; Yucel et al., 2019), but a comprehensive analysis is lacking. Given the active role of metabolism in the proliferation and differentiation of tumor cells and ESCs (Yanes et al., 2010; Lunt and Vander Heiden, 2011), exploring the link between the microenvironment and MuSC metabolism may help identify novel targets to improve both the rate and extent of muscle repair after injury. Additionally, as many skeletal muscle pathologies are linked to a shift in the local metabolic environment (Chi et al., 1987; Joseph et al., 2018), and many metabolic disorders result in impaired skeletal muscle repair (D’Souza et al., 2016; Monaco et al., 2018), it is critical that we understand the link between MuSCs and their local tissue microenvironment.

## A Link Between Metabolism and Skeletal Muscle Regeneration

All cells require energy (in the form of ATP) to sustain the critical enzymatic reactions which support life (Bonora et al., 2012; Petersen and Verkhratsky, 2016), with the loss or significant depletion of ATP resulting in necrosis and cell death (Eguchi et al., 1997). Cellular ATP is primarily generated via either glycolysis or oxidative phosphorylation (OxPhos) in the mitochondria, a process linking the acetyl-coA produced from either glycolysis or fatty-acid oxidation to the tricarboxylic acid (TCA) cycle and the electron transport chain. However, in addition to producing ATP dividing cells must double their cellular content, imposing a large demand for the generation of new biomass in the form of nucleotides for DNA/RNA, amino acids for proteins, and phospholipids for cellular membranes (Lunt and Vander Heiden, 2011). Therefore, it is unsurprising that both the local metabolic environment and innate cell metabolism can dictate processes such as the rate of proliferation and/or differentiation (DeBerardinis et al., 2008; Ryall and Lynch, 2018; Zhu and Thompson, 2019).

In one of the first studies to investigate metabolism and MuSC biology, Rocheteau et al. (2012) observed that MuSCs freshly isolated from uninjured skeletal muscle contained variable levels of mitochondria, with an inverse correlation between mitochondria density and the expression of the transcription factor Pax7. The authors observed that Pax7^Hi^ cells contained the lowest level of mitochondria, while Pax7^Lo^ contained the highest. More recently, quiescent MuSCs have been found to transition between quiescence and an intermediate phase termed G_Alert_, with MuSCs rapidly shifting to this alert phase following injury (Rodgers et al., 2014). Of relevance to the current discussion was the finding that MuSCs in the alert phase were larger and exhibited a greater level of mitochondrial DNA. Whether the Pax7^Lo^ MuSCs identified by Rocheteau et al. (2012) were in the G_Alert_ phase has yet to be confirmed.

In the context of skeletal muscle injury and repair, MuSCs undergo a metabolic switch from fatty-acid oxidation in quiescence to an increased reliance on glycolysis during *in vitro* activation and proliferation (Ryall et al., 2015b). This shift toward glycolysis in activated MuSCs has been confirmed *in vivo* by Pala et al. (2018), who performed an extensive characterization of metabolism in quiescent and active MuSCs and found that the extracellular acidification rate (ECAR, a measurement of glycolytic activity) and oxygen consumption rate (OCR, a measure of OxPhos), was highest in MuSCs isolated from skeletal muscle 3 days post-injury. This peak in metabolic activity occurs during a period of rapid MuSC proliferation (Gayraud-Morel et al., 2009; Quintero et al., 2009; Kimura et al., 2015; Hardy et al., 2016; Xiao et al., 2016). Interestingly, the first 24–48 h of MuSC activation are marked by a significant increase in autophagic flux, with inhibition of autophagy leading to a delay in MuSC activation (Tang and Rando, 2014). The precise role of this acute rise in autophagy, and its importance in terms of MuSC proliferation has yet to be determined.

The peak in ECAR in MuSCs has been observed to decline by day five post-injury, without a concomitant decrease OxPhos, suggesting that a transition toward OxPhos may be required as MuSCs return to a quiescent state (Pala et al., 2018). A similar switch has been observed in many other proliferating cell types including ESCs, hematopoietic stem cells (HSCs), induced pluripotent stem cells (iPSCS) and most notably in cancer cells, and is termed “aerobic glycolysis” or “The Warburg Effect” (Warburg, 1956; Suda et al., 2011; Zhang et al., 2012; Moussaieff et al., 2015).

Professor Otto Warburg first defined the process of aerobic glycolysis in highly proliferative tumor cells, after observing that even in the presence of saturating levels of oxygen, these cells consumed large amounts glucose and extruded lactose (Warburg, 1956). Since this seminal work, researchers have found a link between elevated glucose consumption and cell proliferation in a wide range of cell types including embryonic kidney cells, cancer cells, vascular smooth muscle cells, mesenchymal stem cells, and ESCs (Saki et al., 2013; Han et al., 2015; Shao et al., 2018). While differentiated cells typically convert one molecule of glucose into two molecules of ATP and two molecules of pyruvate which are then used to drive OxPhos in the mitochondria to produce an additional 30–34 molecules of ATP, proliferating cells re-route glycolytic intermediates to drive anabolic reactions and the production of new biomass (Vander Heiden et al., 2009). Under these conditions, each molecule of glucose generates significantly less than two molecules ATP and two molecules of pyruvate. Therefore, proliferating cells must carefully balance their production of biomass with the need for ATP.

Cell division in proliferating cells is achieved via progression through the cell cycle, comprising an initial gap (G_1_) phase where cells double their cellular content, an S phase whereby DNA is replicated, a second gap (G_2_) phase where replicated DNA is checked, and finally mitosis (M phase) where cells undergo division. Importantly, as cell division is a metabolically demanding process several checkpoints exist, and only allow a cell to proceed when certain conditions are met. One such checkpoint exists in the late G_1_ phase where increased glycolytic flux is required prior to the G_1_ to S transition (Kalucka et al., 2015). In addition to ensuring sufficient supply of biomass to dividing cells, this increased reliance on glycolysis during cell-division is also likely a mechanism to reduce the production of reactive oxygen species (ROS) to protect against DNA damage.

While cell-cycle progression is regulated by metabolite availability, the cycle itself can directly regulate the activity of several key metabolic enzymes. In one such study, Wang and colleagues found that cyclin D3 activation of cyclin-dependent kinase 6 (CDK6) phosphorylated and inhibited the catalytic activity of phosphofructokinase 1 (PFK1) and pyruvate kinase M2 (PKM2) (Wang et al., 2017). The inhibition of these two enzymes allowed for the accumulation of glycolytic intermediates and increased flux through the pentose phosphate pathway (PPP) to support nucleotide synthesis.

## Nucleotide Synthesis Through the Pentose Phosphate Pathway

**FIGURE 2:**
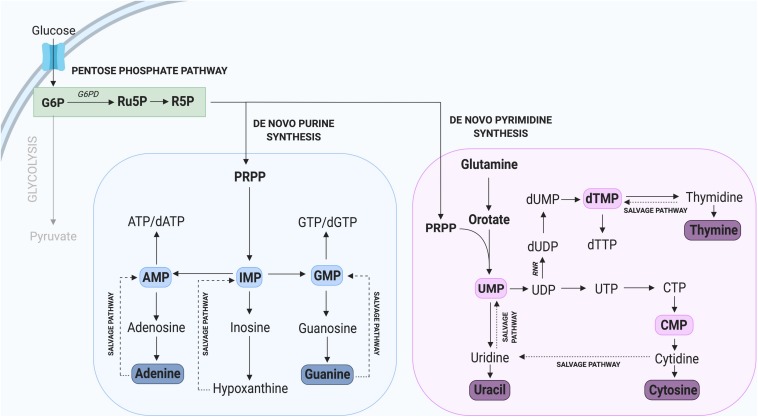
Nucleotide synthesis via the Pentose Phosphate Pathway. Glycolysis contributes to the synthesis of new purine and pyrimidine nucleotides through G6P, which can be converted to the precursor R5P. Not all intermediate steps are shown. AMP, adenosine monophosphate; dATP, deoxyadenosine triphosphate; dGTP, deoxyguanosine triphosphate; dTMP, deoxythymidine monophosphate; dTTP, deoxythymidine triphosphate; dUMP, deoxyuridine monophosphate; GMP, guanosine monophosphate; IMP, inosine monophosphate; UMP, uridine monophosphate.

Nucleotides are essential components of molecules such as ATP, GTP, cAMP, cGMP, and in the synthesis of RNA and DNA (Lane and Fan, 2015), including purines (adenine and guanine) and pyrimidines (cytosine, uracil, and thymine) which differ by the inclusion of either a double carbon and nitrogen ring (purines) or a single carbon ring (pyrimidines). Nucleotide *de novo* generation is achieved via the PPP, one of the first alternate carbon cycles to branch from the main glycolytic pathway and requires simple precursor molecules to be converted to complex nucleic acids (Riganti et al., 2012; Kowalik et al., 2017). In the PPP, glucose-6-phosphate (G6P) undergoes several oxidative carboxylation reactions to form ribose-5-phosphate (R5P) and nicotinamide adenine dinucleotide phosphate (NADPH). R5P serves as a nucleotide precursor, whereas NADPH has a key role in protecting cells from oxidative damage and serves as the major electron donor in many reducing reactions (Meitzler et al., 2014). The flow of glucose into the PPP is first catalyzed by the enzyme glucose-6-phosphate dehydrogenase (G6PD), which irreversibly leads to the oxidative decarboxylation of G6P (Figure 2).

The critical importance of G6PD and the PPP in supporting cell proliferation has been confirmed in several studies demonstrating that its inhibition leads to a significant reduction in tumor and plasmodium cell proliferation (Hu et al., 2015; Xu et al., 2016; Zhang et al., 2017). In contrast, increased activity of G6PD such as that observed in tumor cells, is typically linked with rapid cell proliferation (Du et al., 2013). Interestingly, embryonal rhabdomyosarcoma (ERMS), an aggressive form of cancer involving muscle cells that fail to differentiate, express high levels of *G6PD*. In contrast, following the forced differentiation of these tumorigenic cells, *G6PD* was one of the most highly downregulated genes (Coda et al., 2015). In the context of MuSCs, several whole transcriptome studies in mice have revealed a specific enrichment of *G6pd2* and *G6pdx* in proliferating compared to quiescent MuSCs (Liu et al., 2013; Ryall et al., 2015b). These results strongly support a key role for G6PD in regulating myogenic cell proliferation, likely through provision of new nucleotides.

In addition to *de novo* nucleotide synthesis through the PPP, nucleotides can be generated through recycling or salvage pathways (Figure 2), which predominate during quiescence and differentiation when only low levels of nucleotide synthesis are required (Fairbanks et al., 1995). The nucleotide salvage pathway recycles intermediates derived from the breakdown of DNA and RNA and converts them to purines and pyrimidines. Importantly, the salvage of nucleotides requires only one molecule of ATP per pyrimidine synthesized, compared with seven molecules required by *de novo* synthesis (Nyhan, 2014; Marsac et al., 2019). The core of the purine salvage pathway relies on the regeneration of nucleobases adenine, inosine, and guanine which can be used to generate ATP, IMP, and GTP, respectively. Of these three nucleotides, IMP exhibits the greatest flexibility with the ability to be converted into GMP or AMP when required (Ljungdahl and Daignan-Fornier, 2012; Peifer et al., 2012). This is important during tissue homeostasis and for cells to utilize a low energy pathway to maintain nucleotide levels. This is observed in terminally differentiated neurons, which rely on salvage pathways to maintain nucleotide homeostasis (Fasullo and Endres, 2015).

Having two distinct pathways to synthesize nucleotides (recycling and *de novo*) is an advantage for mammalian cells (Lane and Fan, 2015), as it allows for cells to adapt based on environmental stimuli such as nutrient and/or substrate availability. During periods of cell stress such as limited nutrient availability, cells utilize the salvage pathway to facilitate nucleotide homeostasis. In contrast, cells undergoing rapid proliferation cannot rely solely on *de novo* synthesis, since this pathway is insufficient to facilitate the demand for new nucleotides.

Nucleotide biosynthesis has received scant attention in skeletal MuSCs, but a recent study by Tran et al. (2019) reported on ribonucleotide reductase (RNR) knockout mouse. In this study the authors developed a mouse model with exon 9 of the M1 subunit of RNR flanked by two loxP sites (*Rrm1*^fl/fl^) and bred it with a mouse expressing Cre recombinase under the control of muscle creatine kinase (*Mck*^cre^), with the resulting mouse expressing a truncated and inactive form of RNR in cardiac and skeletal muscle from embryonic day 13 (Tran et al., 2019). As RNR is a key enzyme for *de novo* nucleotide synthesis, its conditional ablation allowed the investigators to study the importance of this pathway in skeletal and cardiac muscle. Importantly, ablation of RNR was found to be lethal within a few days of birth, with a median survival age of 11.5 days and a maximal age of 27 days. In mice that survived to P15–P17, the hearts were found to contain disrupted nucleotide levels with a threefold decrease in dGTP and a twofold increase in dCTP and dTTP compared to wildtype hearts. These results suggest that (in the heart) *de novo* nucleotide synthesis is only critical for the production of dGTP. While not described in detail, the authors found that muscle fibers in the gastrocnemius muscles of knockout mice were less than half the size of those observed in wildtype mice. Additionally, the number of nuclei per fiber was reduced by more than half (Tran et al., 2019). It will be critical in future studies to determine whether a similar defect in dGTP (as observed in cardiac muscle) is observed in skeletal muscle of RNR knockout mice.

## Amino Acid Synthesis Via Glycolysis, the PPP, and the TCA Cycle

Protein accounts for the majority of dry cell mass and is responsible for the formation of key cellular components including antibodies, enzymes, and cell structures (Hosios et al., 2016). Therefore, in addition to nucleotides, there is strong demand for the synthesis of NEAAs during proliferation. In mammalian cells there are nine “essential” amino acids (EAAs, histidine, isoleucine, leucine, lysine, methionine, phenylalanine, threonine, tryptophan, and valine) which cannot be synthesized and must be taken up exogenously. The remaining 11 NEAAs (alanine, arginine, asparagine, aspartic acid, cysteine, glutamic acid, glutamine, glycine, proline, serine, and tyrosine) can be synthesized in the cytoplasm through glycolysis and its sidechains, and in the mitochondria through the TCA cycle.

**FIGURE 3:**
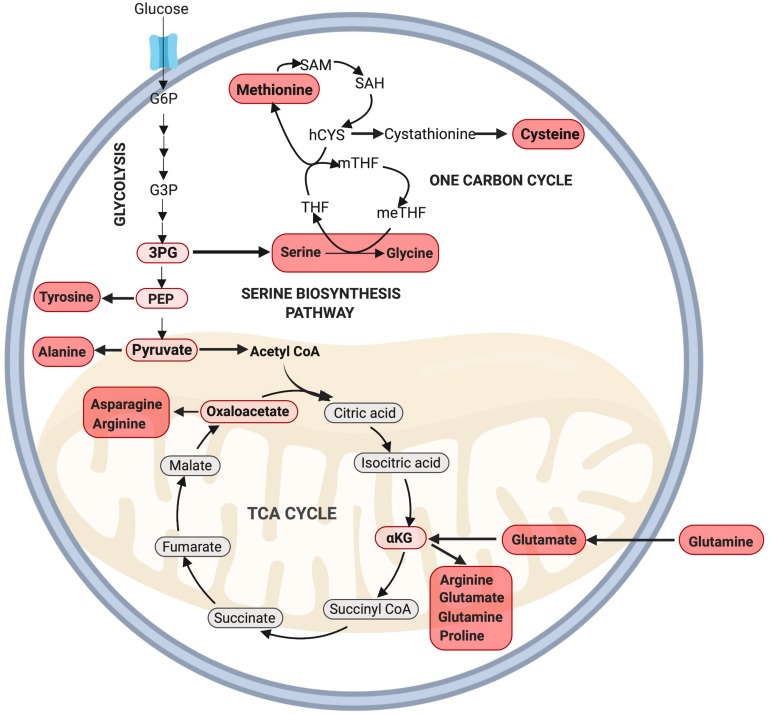
Amino acid synthesis via glycolysis and the TCA cycle. NEAAs can be synthesized through various intermediate metabolites of glycolysis including 3PG, PEP, pyruvate, and the TCA cycle through oxaloacetate and αKG. Note: not all intermediate steps are shown. 3PG, 3-phosphoglyceric acid; hCYS, homocysteine; meTHF, 5,10-methylene THF; mTHF, 5-methyl THF; PEP, phosphoenolpyruvate; SAH, *S*-adenosyl homocysteine; SAM, *S*-adenosylmethionine; THF, tetrahydrofolate.

Besides the generation of ATP, several intermediates of glycolysis can be used to generate amino acids, including 3-phosphoglyceric acid (3PG) and pyruvate (Locasale, 2013). 3PG can contribute carbons to the generation of cysteine, glycine, and serine through the one carbon (1C) cycle while pyruvate can be converted into alanine (Olson et al., 2016; Figure 3). Serine derived from the 1C cycle can combine with the folate cycle to form glycine or it can be utilized in the synthesis of phospholipids as phosphatidylserine (PS) (Glinton et al., 2018). The serine biosynthesis pathway is commonly upregulated in highly proliferative tumors to support growth (Mattaini et al., 2016) and is critical to support MuSC proliferation, as its depletion has been found to prevent MuSCs from transitioning from G1 to S-phase of the cell-cycle (Thalacker-Mercer et al., 2019). Furthermore, Ryall et al. (2015b) have found that multiple enzymes in the serine biosynthesis pathway (including *Phgdh*, *Psat1*, *Psph*, *Shmt2*) are all enriched in proliferating MuSCs in mice. Together, these results provide strong evidence for a key role of serine biosynthesis in regulating MuSC proliferation. Whether this pathway may also play a role beyond the simple provision of NEAAs to dividing cells is an exciting topic deserving of further research.

Recently, the EAA methionine was identified as a powerful anabolic agent capable of regulating cell proliferation. In this study, Walvekar et al. (2018) identified that methionine supplementation alone could increase cell proliferation in yeast cells grown in an amino acid depleted medium. Strikingly, this methionine-dependent increase in proliferation was greater than the anabolic response provided through supplementation of any of the other 18 non-sulfur amino acids. These authors confirmed that methionine increased amino acid synthesis and *de novo* nucleotide synthesis through the PPP and glutamate synthesis pathways (Walvekar et al., 2018). In a related study, Gao et al. (2019) found that dietary methionine restriction alone could significantly reduce tumor growth in mice, and identified a potential mechanism through disruption of *de novo* nucleotide synthesis and the cellular redox balance.

While the importance of methionine in muscle regeneration has yet to be examined, it raises an important question as to whether dietary methionine supplementation alone may improve muscle growth and repair. Interestingly, dietary methionine supplementation in rainbow trout promoted hyperplasia and muscle growth (Alami-Durante et al., 2018), but in chickens only marginal effects on protein synthesis and degradation were reported (Zeitz et al., 2019), indicating that the effect of methionine on skeletal muscle growth and regeneration requires further investigation.

During the proliferating phase of an immortalized murine myogenic cell line (C2C12 myoblasts), glutamine is the second most highly consumed nutrient besides glucose and plays a key role in anaplerosis (Hosios et al., 2016), and amino acid and nucleotide biosynthesis (DeBerardinis et al., 2007). Following transport into the cell, glutamine undergoes a deamination reaction catalyzed by the enzyme glutaminase to produce glutamate. This process, known as glutaminolysis, is critical for cell proliferation (Choi and Park, 2018). Glutamate can then be either converted into glutathione or α-ketoglutarate via oxidative deamination to supply the TCA cycle. Unsurprisingly, glutamine is added to cell culture media to support cell growth as its deprivation leads to cell cycle arrest at the S phase (Gaglio et al., 2009).

## Phospho/Lipid Synthesis Via Glycolysis and the TCA Cycle

Lipids constitute key components of the cellular plasma membrane, act as an energy source/store, and play key signaling roles through the production of hormones (Watt and Hoy, 2011; Hubler and Kennedy, 2016; Yao et al., 2016). Therefore, lipid metabolism is another critical process for rapidly proliferating cells, including cancer cells and neural stem/progenitor cells (NPSCs) which exhibit elevated exogenous lipid uptake and *de novo* lipid synthesis (Natter and Kohlwein, 2013; Knobloch, 2017; Zhao et al., 2017; Yi et al., 2018).

Lipids can be divided into several separate classes based on their chemical structure and properties, with each having distinct roles within cells. The major classes of lipids incorporated into mammalian cells comprise phosphatidic acid (PA), phosphatidylinositol (PI), PS, phosphatidylethanolamine (PE), phosphatidylcholine (PC), phosphatidylglycerol (PG), and cardiolipin (CL; Calzada et al., 2016). Other lipid classes include (but are not limited to) cholesterols, sphingomyelins, cerebroside, gangliosides, phospholipids, and triacylglycerols (TAGs). Importantly, many of these lipid classes have previously been demonstrated to influence rates of cellular proliferation and differentiation of myogenic cells (Mebarek et al., 2007; Gangoiti et al., 2012). For example, Mebarek et al. (2007) found that inhibition of ceramide synthesis in immortalized rat myogenic cells (L6 myoblasts) led to an increase in the rate of differentiation through the upregulation of phospholipase D, an enzyme responsible for the generation of PA. Conversely, addition of exogenous ceramides resulted in a reduction in the expression of the transcription factor *myogenin*, a key regulator of myoblast differentiation (Mebarek et al., 2007). Similarly, ceramides inhibit anabolic growth in mature skeletal muscle through the inhibition of IGF-1/Akt and mTORC signaling (Akhmedov and Berdeaux, 2013; Hsieh et al., 2014). Interestingly, ceramide-1-phosphate (C1P, derived from ceramide) can induce proliferation of C2C12 myoblasts through increased Akt and ERK1/2 signaling (Bernacchioni et al., 2018). In this manner, ceramide can both inhibit myogenic differentiation and, following conversion to C1P, promote proliferation. However, it is important to note that ceramide itself inhibits proliferation, highlighting the complex nature of lipid signaling (Faustino et al., 2008).

In addition to conversion into C1P, ceramide can also be reversibly converted into sphingosine. Both sphingosine and ceramide are negative regulators of cell growth, and have been linked to cell cycle arrest and apoptosis (Woodcock, 2006; Kanno et al., 2014). Similar to ceramide, sphingosine can be phosphorylated to form S1P, which in C2C12 cells is critical for both the inhibition of proliferation and initiation of differentiation (Donati et al., 2004). In contrast to these findings in C2C12 cells, Calise et al. (2012) found that S1P supplementation stimulated proliferation in primary mouse MuSCs. These authors attributed the discrepancy in their results to differences in S1P receptor type availability between C2C12 cells and primary MuSCs (Becciolini et al., 2006; Calise et al., 2012).

In addition to regulating proliferation and differentiation, sphingomyelin levels change following activation, as quiescent MuSCs exhibit high levels of sphingomyelin within the plasma membrane, which subsequently drop after activation (Nagata et al., 2006). These results highlight the importance and complexity of the ceramide/S1P axis in regulating MuSC proliferation and differentiation during regeneration.

Similar to nucleotides and amino acids, proliferating cells can meet the demand for new lipids by utilizing lipids in the local extracellular environment or performing *de novo* lipid biosynthesis from glycolytic intermediates The *de novo* synthesis of all phospholipids (with the exception of PA), requires pyrimidine nucleotide cytidine triphosphate (CTP), which is synthesized in the PPP. Interestingly, CTP synthase, which catalyzes the rate limiting step of *de novo* CTP synthesis, is upregulated in many cancer lines (Williams et al., 1978), and its inhibition reduces cell proliferation through impaired nucleotide and phospholipid synthesis. Therefore, proliferating cells require a coordinated effort of nucleotide synthesis for DNA and phospholipids (Verschuur et al., 2000).

**FIGURE 4:**
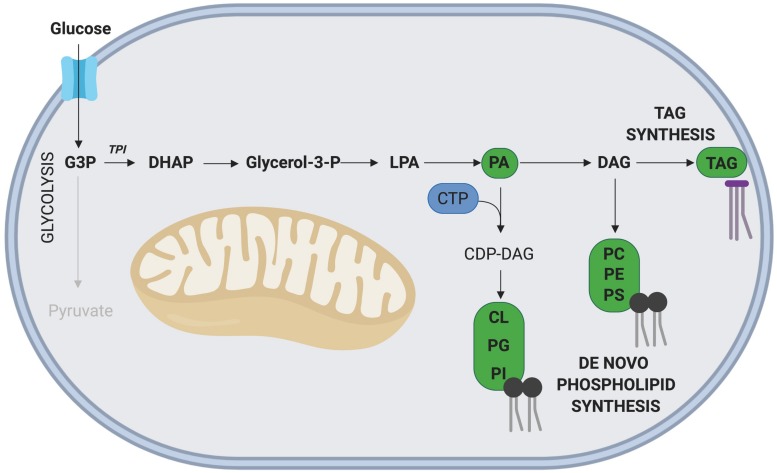
Phospholipid and TAG synthesis via G3P. The glycolytic intermediate G3P, can contribute to phospholipid and TAG synthesis following its initial conversion to DHAP. The nucleoside CTP is required for synthesis of all phospholipids except phosphatidic acid (PA). Key metabolites of this side pathway include PA and diacylglycerol (DAG). Note: not all intermediate steps are shown. CDP-DAG, cytidinediphosphate-diacylglycerol; CL, cardiolipin; LPA, lysophosphatidic acid; PC, phosphatidylcholine; PE, phosphatidylethanolamine; PG, phosphatidylglycerol; PI, phosphatidylinositol; PS, phosphatidylserine.

Glyceraldehyde-3 phosphate (G3P), an intermediate metabolite of glycolysis, is intricately involved in the *de novo* synthesis of phospholipids and TAGs (Alves-Bezerra and Gondim, 2012; Figure 4). In this pathway, G3P is first converted into dihydroxyacetone phosphate (DHAP), a reaction catalyzed by the enzyme triosephosphate isomerase (TPI), with TPI1 expression correlated with increased rates of proliferation in gastric cancer cells (Chen et al., 2017). Interestingly, overexpression of *TPI1* in hepatocellular carcinoma cells impairs proliferation (Jiang et al., 2017), suggesting the role of TPI may be cell type specific. While the role of *Tpi1* in MuSC proliferation has not been directly assessed, several transcriptomic studies conducted on freshly isolated and proliferating MuSCs have revealed that elevated *Tpi1* expression in proliferating MuSCs (Ryall et al., 2015b). Further research is required to determine the role of Tpi1 in myogenesis and skeletal muscle regeneration.

In another C2C12 based experiment, Lee et al. (2009) demonstrated that supplementing proliferating cells with mono-unsaturated FAs, n-6-polyunstaurated FAs, linoleic acid, gamma-linoleic acid and arachidonic acid all enhanced proliferation. Exogenous arachidonic acid has also been found to promote myoblast differentiation through its conversion to prostaglandin E_2_ (PGE2) in a COX-2 dependent-manner (Leng and Jiang, 2019). Of interest, PGE2 has been found to be rapidly synthesized and released into the local muscle microenvironment following damage. Ho et al. (2017) demonstrated that PGE2 is required for successful regeneration, as inhibition of PGE2 synthesis led to impaired MuSC proliferation and weakened muscles.

In contrast to mono-unsaturated fatty-acids, the saturated fatty acid palmitate significantly inhibited C2C12 myoblast proliferation through a reduction in both cyclin A and cyclin D1, while promoting differentiation and increased myotube width (Grabiec et al., 2015). Taken together, these results highlight the importance of a regulated role for fatty acids, as the dysregulation or excessive accumulation of fatty acids in the MuSC microenvironment may negatively affect skeletal muscle regeneration. This is evident in models of diabetes mellitus and obesity, which are characterized by excess fatty acids in the microenvironment, insulin resistance and impaired glucose tolerance. Both models display impaired muscle regeneration following injury (Hu et al., 2010; Nguyen et al., 2011; Akhmedov and Berdeaux, 2013; D’Souza et al., 2015; Xu et al., 2018). In one study, MuSC activation and proliferation was impaired in insulin resistant *ob/ob* mice, and in another, myotube maturation was delayed (Hu et al., 2010; Nguyen et al., 2011). Similar to that observed for nucleotides, under nutrient-rich conditions, mammalian cells tend to utilize *de novo* lipid synthesis for cellular proliferation (Palm and Thompson, 2017), but this has yet to be confirmed in MuSCs.

## Conclusion

While metabolism has previously been thought to play a passive role in myogenesis, it is now established as a key regulator of both cell state and lineage progression. When MuSCs undergo rapid proliferation, efficient carbon routing through glycolysis (including its side branches) and the TCA cycle is required for the generation of precursors such as nucleotides, amino acids and lipids/phospholipids. In addition, an adequate supply of nutrients or precursors within the MuSC microenvironment is critical for these metabolic pathways to proceed. Many studies have demonstrated the regulatory effects of various metabolites on MuSCs and other proliferating cell types *in vitro* (either through the supplementation or deprivation), highlighting the importance of a tightly regulated metabolic microenvironment. However, metabolism and nutrient availability during regeneration remains an understudied topic *in vivo*, with many of these effects yet to be confirmed in regenerating skeletal muscle. Further RNAseq studies examining the expression of genes encoding for enzymes in these metabolic pathways combined with carbon-labeled flux analysis will help identify critical genes and/or metabolites which regulate these processes.

A better understanding of how the local metabolic microenvironment may regulate MuSC biology has important application for a broad range of fields, including synthetic biology studies focused on volumetric muscle loss, regenerative medicine and stem cell based therapies, agricultural research attempting to maximize protein yield and even in the developing field of cellular agriculture where researchers are attempting to generate cultivated meat. Together, the studies discussed in this review highlight an important role for metabolism in MuSC biology, particularly in the regulation of proliferation.

## Author Contributions

JN and JR conceived the topic for review. JN, JC, GL, and JR wrote the review.

## Conflict of Interest

The authors declare that the research was conducted in the absence of any commercial or financial relationships that could be construed as a potential conflict of interest.
